# Gaze tracking accuracy in humans: One eye is sometimes better than two

**DOI:** 10.3758/s13428-018-1135-3

**Published:** 2018-10-22

**Authors:** Ignace T. C. Hooge, Gijs A. Holleman, Nina C. Haukes, Roy S. Hessels

**Affiliations:** 1 Experimental Psychology, Helmholtz Institute, Utrecht University, Utrecht, The Netherlands; 2 Developmental Psychology, Utrecht University, Utrecht, The Netherlands; 3 Utrecht University Honours College, Utrecht, The Netherlands

**Keywords:** Eye tracking, Data quality, Accuracy, Precision, Monocular, Binocular

## Abstract

Most modern video eye trackers deliver binocular data. Many researchers take the average of the left and right eye signals (the version signal) to decrease the variable error (precision) up to a factor of $\sqrt {2}$. What happens to the systematic error (accuracy) if the left and right eye signals are averaged? To determine the systematic error, we conducted a calibration validation in two experiments (*n*= 79 and *n* = 64). The systematic error was computed for the left eye, right eye, and version signals separately. In respectively 29.5 and 25.8% of the participants, the systematic error of a single eye signal was lower than that of the version signal at the cost of a higher variable error. If a small variable error is desirable, and the difference between the left and the right eye is not the topic of study, one should average position data from the left and the right eye (in other words, use the version signal). If a small systematic error is desirable, one should use the signal (from left eye, right eye or version) that delivers the best accuracy. In the latter case, this may cause worse precision than that of the version signal.

## Introduction

Eye movements can be measured both binocularly (in two eyes) and monocularly (in one eye), but when to conduct a binocular measurement or not is not always self-evident. For example, if eye tracking is conducted with a scleral coil, which is an invasive and uncomfortable method, the measurement is usually restricted to one eye to limit the nuisance for the participant (Frens, Van Opstal, & Van der Willigen, 1995; Hooge & Erkelens, 1996). Using two coils usually only occurs when the goal is a binocular measurement (Collewijn, Erkelens, & Steinman, 1988; Hooge & Van den Berg, 2000). In video-based eye tracking, this precondition and limitation does not exist because the measurement is not invasive. However, there is another bottleneck to consider, namely that of the cost of some devices. A binocular eye tracker may be more expensive than a monocular one because some are equipped with two cameras instead of one. Therefore, some older tower-mount video eye trackers such as the Senso Motoric Instruments (SMI) iView X Hi-Speed 240 were available in monocular and binocular versions. The same holds for eye-tracking glasses, the monocular ones (e.g., pupil-labs) are usually cheaper than binocular ones. The majority of the modern remote eye trackers (Tobii, SR-Research and SMI) are binocular and deliver left eye and right eye-tracking data separately. There are exceptions, the SMI REDm measures binocularly but the standard data file contains monocular eye-tracking data (this is probably the *version* signal, the average of the left and the right eye signal).

Currently, researchers have the freedom to use eye-tracking data from the left eye (LE), the right eye (RE) or to use data from both eyes. Some researchers only use data from one eye, even if they have data from two eyes (Vlaskamp, Over, & Hooge, 2005). Tatler (2007) also used data from one eye but chose the signal for the eye that produced the better spatial accuracy as determined using the calibration. Eye-tracking data from the two eyes are required if one wants to study binocular coordination (Liversedge, White, Findlay, & Rayner, 2006; Nuthmann & Kliegl, 2009). However, often the LE and RE signals are averaged; we will refer to this signal as the version signal (VS).^1^ Using the VS signal has an advantage. The variable error of the VS signal should, due to the square root law, be a factor of $\sqrt {2}$ smaller than the variable error of a single eye signal. Using the VS signal may help to increase the signal-to-noise ratio in data with low data quality such as eye-tracking data recorded from infants (Hessels, Hooge, & Kemner, 2016).

Throughout this article, we will use the term *systematic error* instead of accuracy and the term *variable error* instead of precision for semantic reasons. We find it counterintuitive that low precision is characterized by a large variable error (often operationalized by RMS sample-to-sample deviation) and that high accuracy is characterized by a small systematic error (often operationalized as the distance between a fixation target and a fixation location under the assumption that the target is fixated by the participant). Averaging the coordinates from both eyes only makes sense if no systematic error (the difference between reported gaze location and actual gaze location) is introduced by using the VS signal instead of the LE or RE signals. However, there are clear cases when averaging can introduce a systematic error. For example, in individuals with disturbed binocular alignment, such as convergence insufficiency (Thorn, Gwiazda, Cruz, Bauer, & Held, 1994; Van Leeuwen, Westen, van der Steen, de Faber, & Collewijn, 1999), averaging LE and RE signals may increase the systematic error in the VS signal. We are not the first to tackle this problem. In an interesting article by Cui and Hondzinski (2006), the authors claimed that using the version signal instead of the signal of a single eye results in a smaller systematic error. In their experiment, they determined the systematic error in eye-tracking data of six participants with a right dominant eye. To do so, they used an ASL Model 501 head-mounted mobile eye tracker that measures at 60 Hz. However, the conclusions achieved with a mobile eye tracker cannot simply be generalized to the eye-tracking data of a modern remote eye tracker. For example, the systematic error of a mobile eye tracker contains a component, the so-called parallax error, that is not present in the eye-tracking data of a remote eye tracker. Li, Babcock, and Parkhurst (2006) wrote: “This is due to that fact that the scene camera is not in the same optical path as the tracked eye” (p. 98).

To our surprise, there are not many studies of the systematic and the variable errors of binocular eye-tracking data. However, there is a study by Svede, Treija, Jaschinski, and Krumina (2015) about the effect of the calibration method on systematic errors. They wrote: “the objective fixation disparity differs depending on whether the calibration is performed monocularly or binocularly, and this difference depends on the individual’s fixation disparity” (p. 13). Fixation disparity refers to the distance on the screen between the fixation positions of the left and the right eyes during fixation of a particular location on screen. Both monocular and binocular calibration methods have their advantages and disadvantages. In a monocular calibration procedure, the eye tracker is calibrated for each eye separately with monocularly presented calibration markers. According to Svede et al., (2015), monocular calibration is preferred if the goal of the eye-tracking study concerns fixation disparity (such as e.g., Liversedge et al., 2006). In a binocular calibration procedure, both eyes look at the calibration markers at the same time. This is the standard calibration method used in most eye tracking studies. A disadvantage of the binocular calibration is that if the participant shows fixation disparity during binocular viewing, this fixation disparity is not present anymore in the calibrated eye tracking data (due to the binocular calibration). Most corporate eye trackers do not allow for the choice between a monocular and a binocular calibration procedure. The standard calibration mode is binocular. Researchers who want more calibration options for their corporate eye tracker will have to implement these themselves. A recent article provides the eye-tracking community with a software toolkit that allows for both monocular and binocular calibration with the Tobii EyeX (Gibaldi, Vanegas, Bex, & Maiello, 2017).

In the current study, we will restrict ourselves to investigate eye-tracking signals calibrated with a binocular procedure, which is the most common method, although it is not necessarily the best (Gibaldi et al., 2017; Svede et al., 2015). Many users of binocular eye trackers are not interested in binocular control. Our goal is to provide these users with a decision rule to motivate the use of the left eye, the right eye or the version signal for their analysis. We measured the systematic and variable errors of a Tobii TX300 high end remote eye tracker in two experiments (with 79 and 64 naïve participants, respectively). We compared the systematic and variable errors of the single eye signals to the version signal. Our expectation is that the systematic error in the single eye signal may be smaller than in the version signal, because we measured in a broader population than Cui and Hondzinski (2006). In such a broad population there is a chance that participants have disturbed binocular alignment; Stidwill (1997) estimated the prevalence of binocular vision anomaly to be 5% in the general population. In addition to the previous, in a broader population we may encounter all kinds of problems (glasses, make-up etc.) that may affect the quality of the eye-tracking data of the separate eyes in different ways. Due to the square root law, we also expect a higher variable error when using the eye tracking signal from a single eye. We will discuss situations in which a smaller systematic error or a smaller variable error is desirable.

## Method

We have conducted two experiments. We will refer to the first as SF (Smartass Festival, the literal translation of “Betweter Festival”, a cultural and science festival organized in Tivoli Vredenburg, a concert venue in the center of Utrecht) and to the second as R038 (referring to room 0.38 of the Ruppert building at Utrecht University; a large computer room meant for teaching).

### Participants

In the SF experiment, we recorded eye movements of 82 visitors of the festival. We included data from 79 participants (61% female, age ranged from 19 to 70 years, with a mean of 31.7 years and standard deviation of 11.7 years). We excluded data from three participants because we could not calculate the systematic error in at least seven of the nine validation trials. The room where we conducted the experiment was noisy with moderately loud music and talking. In the R038 experiment, we measured eye movements from 65 undergraduate psychology students involved in a programming course. We included data from 64 participants (58.7% female, age ranged from 19 to 44 years with a mean of 21.8 years and standard deviation of 3.4 years). Data of one participant was excluded because of severe nystagmus. He took part in the experiment, as he was interested in having his eye movements measured. The location of the R038 experiment was less noisy than the Smartass Festival, although at least 100 students and ten teachers were present in the room during the measurements.

### Apparatus

**Fig. 1:**
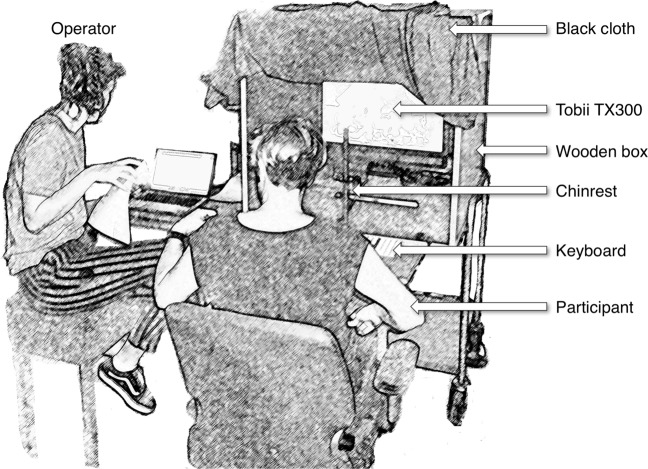
The setup consists of a wooden box placed on a cart. The box contains a chin-rest and Tobii TX300 eye tracker. We attached a small desk with a keyboard to the front of the wooden box. In this illustration, the participant receives instructions from the operator. When the operator is ready, the participant will take place in the chin-rest and the black cloth is draped over the participant

Eye movements of the right and the left eye were measured at 300 Hz with the Tobii TX300. The eye tracker was placed in a mobile booth (see Fig. 1). The screen was located at a distance of 65 cm from the participant, has a resolution of 1920 × 1080 pixels, and measures 51.1 cm (42.9^∘^) × 28.7 cm (24.9^∘^). During the experiment, the participants were seated underneath a black cloth to make sure that the lighting conditions were the same for each participant. We used a chin- and forehead rest to prevent head movements. Head movements and non-optimal head orientations (e.g., tilted) and positions (e.g., edge of the eye tracker head box) are known to increase the systematic error (Hessels, Cornelissen, Kemner, & Hooge, 2015; Niehorster, Cornelissen, Holmqvist, Hooge, & Hessels, 2018).

### Procedure

**Fig. 2:**
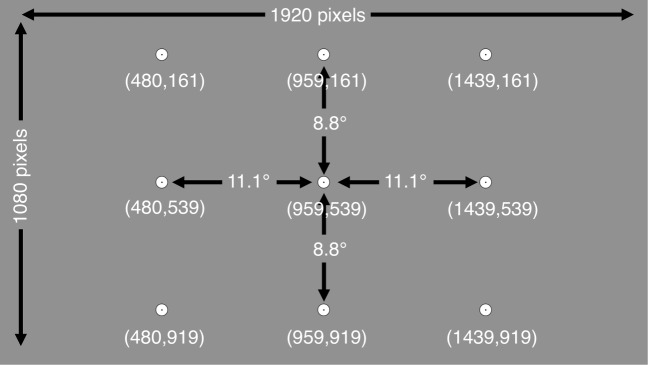
The fixation markers (diameter 0.87^∘^) of the separate calibration validation displays combined in one figure. The *numbers between parenthesis* denote the pixel coordinates of the centers of the fixation markers

To estimate the systematic and variable errors, we conducted a separate calibration and a separate calibration-validation procedure. The experiment started with a standard binocular nine-point calibration (extending 22.2^∘^ by 17.6^∘^), followed by the presentation of one or more movie clips. The total duration was 35 s for the SF and 240 s for the R038 experiments. Hereafter, the calibration-validation procedure was conducted. In the calibration-validation procedure, the participants were presented with nine subsequent screens that each contained one fixation marker (diameter 37 pixels/0.87^∘^). Together, the nine fixation markers formed a virtual nine-point rectangular grid (see Fig. 2), extending 22.2^∘^ × 17.6^∘^. When presented with a calibration-validation screen, the participants were asked to fixate the fixation marker carefully and press the space bar while they were fixating the marker. Following the space bar press, the next validation screen was presented. This is known as a participant-controlled method and delivers higher data quality as compared with the system- and operator-controlled methods (Nyström, Andersson, Holmqvist, & Van de Weijer, 2013).

### Post-processing and data analysis

In order to calculate the systematic error and the variable error, we performed the following steps:

1. To construct the VS signal, LE, and RE signals were averaged per sample.
2. We classified fixations in the LE, RE, and VS signals using the algorithm described in Hooge and Camps (2013). Fixations under 60 ms were discarded from the analysis.
3. The last fixation occurring in each of the nine validation trials was selected. From the gaze coordinates during this fixation the systematic error was calculated. This was done by first calculating the average fixation location, and subsequently calculating the distance between the average fixation location to the validation target.
4. The variable error was determined by computing the RMS sample-to-sample deviation during the selected fixation.
5. Only when the error could be obtained in at least seven of the nine validation trials we calculated the mean systematic and the mean variable errors for each subject. This was done by averaging the errors for the individual validation trials.

### Ethics statement

The study adhered to the Declaration of Helsinki (World Medical Association, 2013) and ethical approval of the research proposal for the SF experiment was received after examination by the Faculty Research Ethics Board of the Faculty of Social and Behavioural Sciences of Utrecht University (FETC17-097-Hooge). Written informed consent was obtained from each participant prior to the start of the study. Participants in the R038 experiment received 0.5 so-called “participant hours”, of which they have to complete 12 during their studies.

## Results

**Fig. 3:**
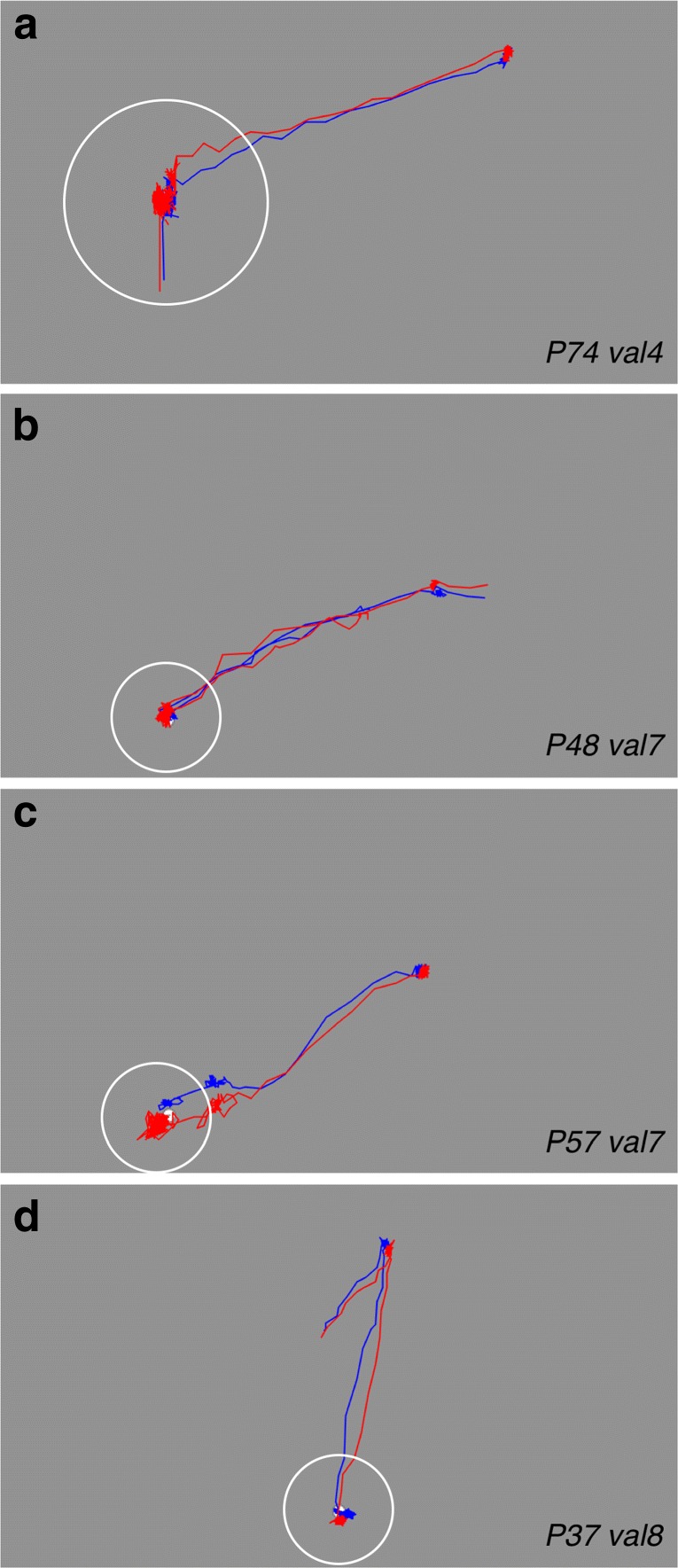
Examples of fixations used for computation of the systematic and variable errors (this figure is at the same scale as Fig. 2). *Blue* denotes signals of the left eye and *red* denotes signals for the right eye. The centers of the *white circles* contain the fixations that were used for calculation of the systematic and the variable errors. The calibration was self-paced, some participants only made saccades from one fixation point to the next one in the next display (panel **a**), other participants did not necessarily make saccades from one calibration point to another, they may have fixated anywhere on the screen before they fixated the calibration target (as in **b**, **c**, and **d**). Panels **a** and **b** show examples in which left and right eye signals overlap. Panels **c** and **d** contain examples that show larger systematic errors for the left eye (**c**) and right eye (**d**). Variable error is larger in panels **a** and **c** compared to panels **b** and **d**

We measured the systematic error in two calibration validation experiments (SF and R038). Figure 3 shows examples of raw eye-tracking data. Panels a and b show overlapping LE and RE signals. In panels c and d, the LE and RE signals do not overlap. In our datasets it occurred that the systematic error of LE signal was larger than that of the RE signal (panel b) or vice versa (panel c).

**Fig. 4:**
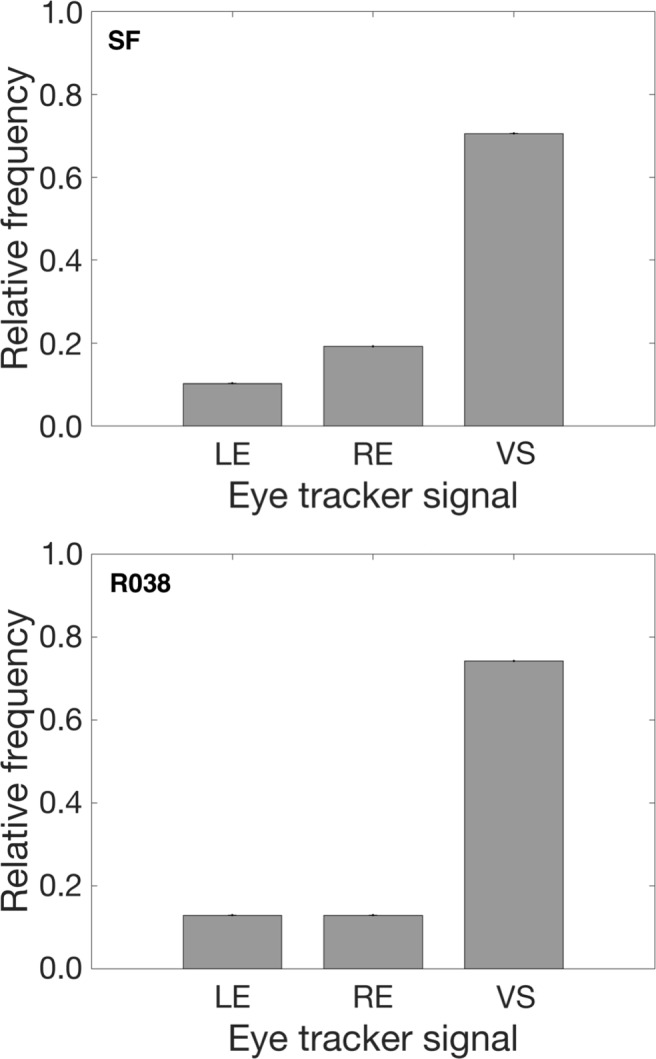
The relative frequency of participants in which the LE, the RE or the VS signal yielded the smallest systematic error. The *top panel* denotes results from the SF experiment (*n*= 79). The *bottom panel* denotes results from the R038 experiment (*n* = 64). In most participants, the VS signal yielded the smallest systematic error, however in 29.5% (SF) and 24.5% (R038) of the participants, the LE and RE signals had smaller systematic errors than the VS signal

Figure 4 shows the relative frequencies of the smallest systematic error occurring in the LE, RE, or VS signals. In most participants, the version position signals yielded the smallest systematic error compared to the errors of the left and the right eye signals separately (Fig. 4, Top panel SF 70.5%; Bottom panel R038 74.2%). However, this means that the use of the version signal (as many researchers do and some eye trackers have as a default setting) is suboptimal in 24.5% (R038) to 29.5% (SF) of the participants. By definition, choosing the eye-tracking signal that has the smallest systematic error for each participant will deliver a smaller systematic error over all participants than choosing the same signal for each participant. The magnitude of the maximal decrease in systematic error by applying this rule was determined by computing the systematic error averaged over all participants in four different ways (LE, RE, VS, and the minimum [MIN] of the LE, RE, or VS signals).

In order to ascertain whether the systematic errors differed significantly between the signals and between the two experiments, we conducted a mixed-design ANOVA with experiment (SF and R038) as a between-subjects factor and signal (LE, RE, VS, and MIN) as a within-subjects factor. The alpha level was set at 0.05. The overall systematic error was not significantly different between experiments (F(1,136) = 3.83, *p*= 0.052). As the assumption of sphericity was violated for the within-subjects factor of signal, Greenhouse–Geisser corrections were used. The systematic error was significantly different between the four signals (F(1.39,189.51) = 35.21, *p*< 0.001). Post-hoc *t* tests with Bonferroni-corrected *p* values revealed that the systematic error for the LE and RE signals were significantly larger than the systematic error for the VS and MIN signals (all *p*< 0.001). The systematic error for the VS signal was significantly larger than for the MIN signal (*p*= 0.015). The systematic errors for the LE and RE signals did not differ significantly (*p*= 1). There was no significant interaction between experiment and signal (F(1.39,189.51) = 1.18, *p*= 0.295). This means that the relative pattern of the systematic error in the SF experiment is the same as in the R038 experiment.

**Fig. 5:**
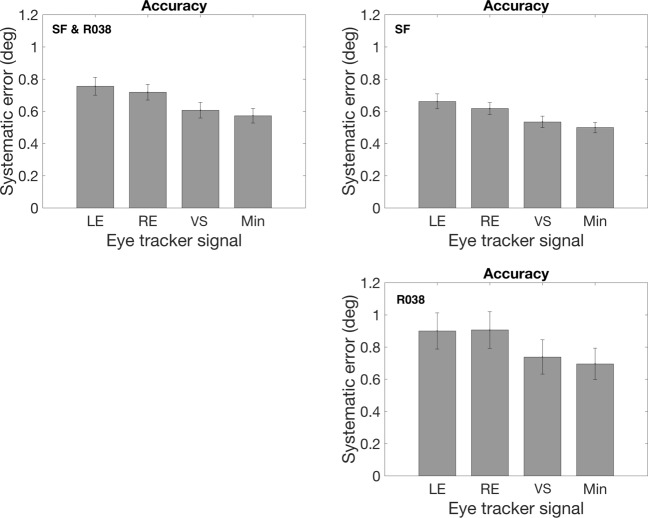
The systematic error. *Left* (SF and R038), *right* (SF) and *bottom* (R038) *panels* depict mean systematic error calculated based on the left eye (LE), right eye (RE), version (VS) and minimum of LE, RE and VS signals. *Error bars* denote standard error of the mean

Figure 5 depicts systematic errors of the four signals (LE, RE, VS, and MIN) for the entire dataset and for the R038 and SF experiments separately. In the SF experiment, choosing the signal with the smallest systematic error instead of using the version signal delivered a systematic error that was 7.4% smaller. In the R038 experiment, choosing the signal with the smallest systematic error delivered a systematic error that was 9.5% smaller. The systematic error reduction is about similar in both experiments, which suggests that it makes sense to select the eye-tracker signal with the lowest systematic error.

**Fig. 6:**
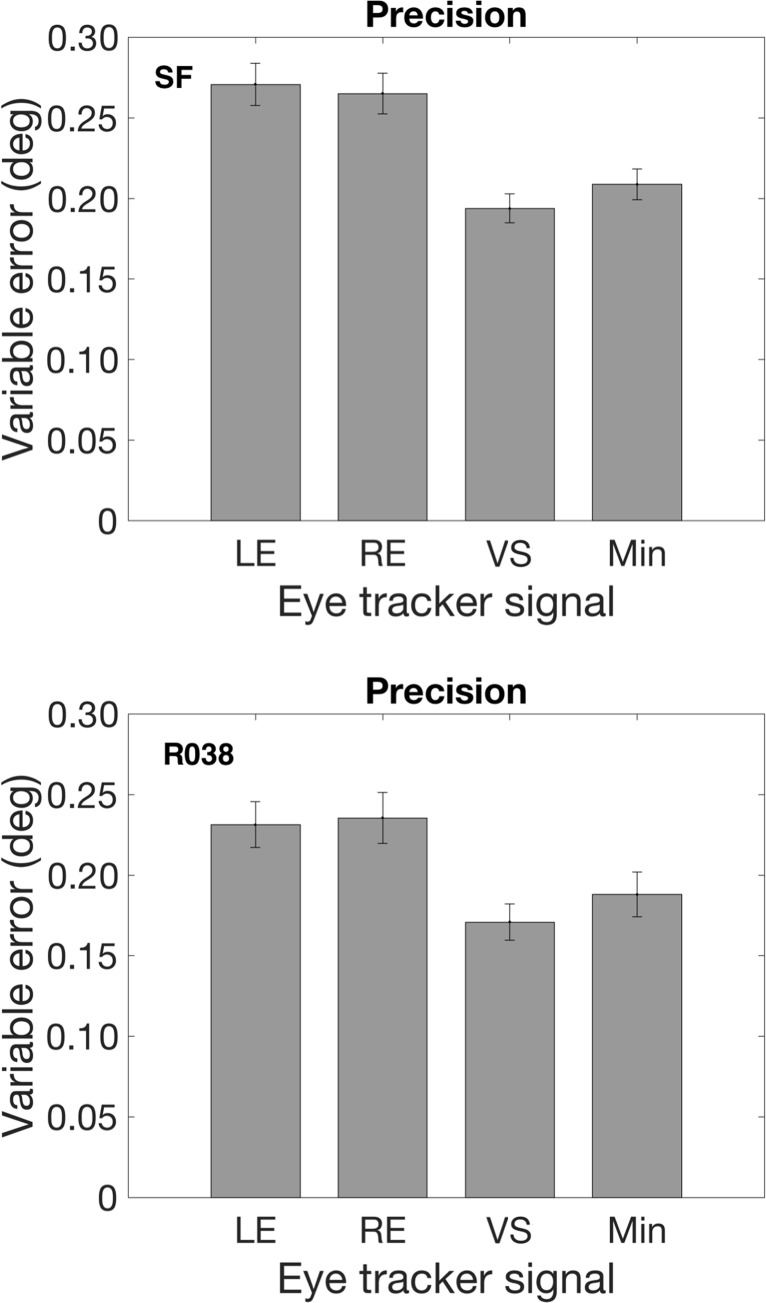
*Top* (SF) and *bottom* (R038) *panels* show variable error for LE, RE, VS, and minimum of LE, RE, and VS signals with the smallest systematic error. *Error bars* denote standard error of the mean

Minimizing the systematic error by choosing the signal with the smallest systematic error may cause a higher variable error. This is indeed the case; Fig. 6 shows that the variable error of the version signal is smaller than the variable error of the signal with the smallest systematic error. In the SF experiment, the variable error was 6.8% higher and in the R038 experiment the variable error was 6% higher.

The values for the decrease in the systematic error, and the increase in the variable error here presented, may be specific to the eye tracker, the experimental conditions and the populations we employed. Values may be different for different eye trackers (for example because some manufacturers apply filtering to the raw data to decrease the variable error), different experimental conditions, or different participant groups. However, we presented the values here to illustrate the gain that may be achieved in two separate experiments.

**Fig. 7:**
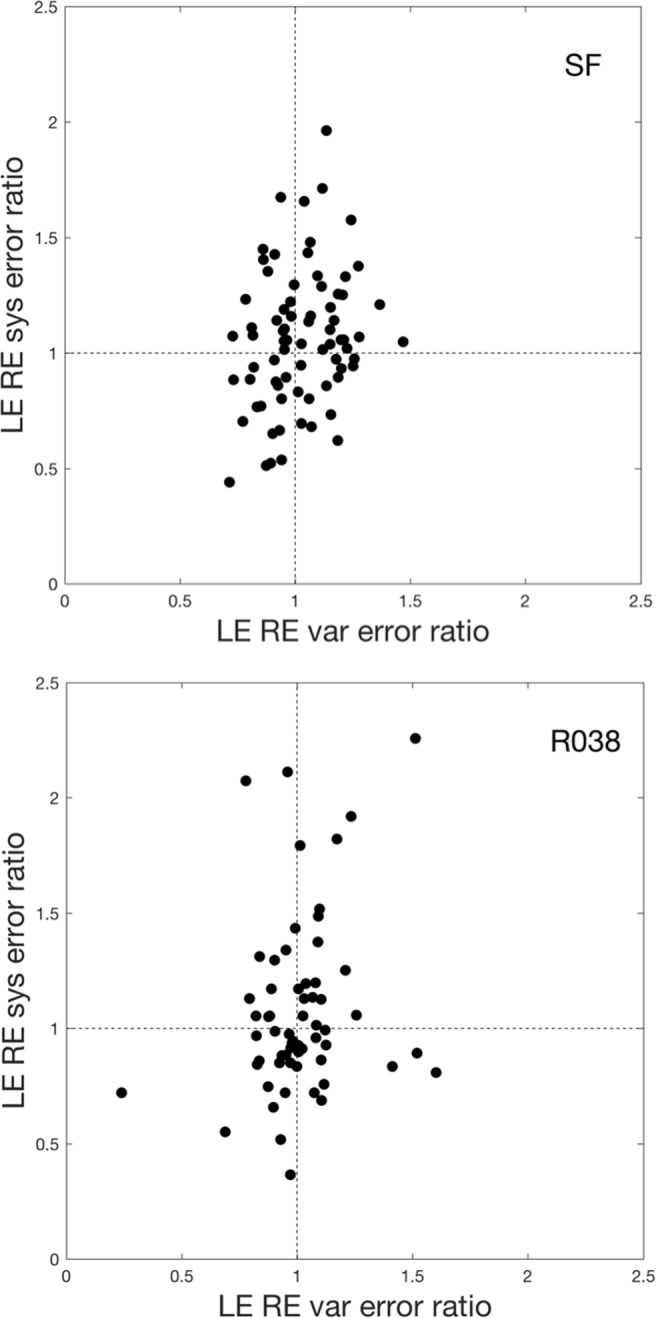
Relation between data quality in the left and right eye. The ratio of LE and RE systematic error vs. the ratio of LE and RE variable error. The *top panel* represents data from the SF experiment, and the *bottom panel* represents data for the R038 experiment. Each *dot* shows error ratios for one participant. Data for two participants of the R038 experiment were not shown here because their LE-RE systematic error ratio exceeded the maximum of the *y*-axis range

With more knowledge of the systematic and variable errors in the eye-tracker signals of the two eyes, and the relation between the two, one may construct more comprehensive rules for selecting which eye-tracker signal to analyze. For example, is it the case that when the systematic error in the LE signal is larger than in the RE signal, that the variable error is also larger for the LE signal? Or are the two independent? Two potentially interesting candidates to compare the LE and RE eye-tracker signals are the ratios of the systematic and the variable error. Figure 7 shows these ratios and it is clear that in our two experiments (1) variable and systematic errors are mostly decoupled and (2) the range of the systematic error ratio is larger than the range of the variable error ratio.

## Discussion

In this study, we investigated the systematic error of the left and the right eye and version signal separately, in two experiments with 79 and 64 participants. To ensure that we have a measurement that is representative for high-quality eye-tracking research, we first relate the data quality achieved here with the data quality as reported by the eye-tracker manufacturer.

### Data quality of the present study

For the TX300, Tobii specifies a systematic error of 0.5^∘^ (monocular) and 0.4^∘^ (binocular). The values for the systematic error from the SF experiment are close to the ideal (Tobii) values. We obtained 0.64^∘^ (monocular) and 0.53^∘^ (binocular). In the R038 experiment, the values for the systematic error are higher (0.90^∘^ monocular and 0.74^∘^ binocular).

Tobii reports a variable error of 0.22^∘^ (monocular) and 0.14^∘^ (binocular). In the SF experiment we found 0.27^∘^ (monocular) and 0.19^∘^ (binocular) and in the R038 experiment 0.23^∘^ (monocular) and 0.17^∘^ (binocular). Here the values for the R038 experiment are better and very close to the ideal (Tobii) values. Considering that we have conducted our experiments with a mobile booth in noisy rooms outside the laboratory and in the SF-experiment with participants from the general population, we are not dissatisfied with these values for our systematic errors.

### The systematic error

Cui and Hondzinski (2006) state that “Two eyes are better than one” (p. 257) if it concerns the accuracy of human eye tracking. In this study, we examined whether averaging the left and right eye signals leads to smaller systematic errors compared to the signals from a left or right eye. This does not appear to be the case. In 25 to 30% of the participants, the systematic error in the eye tracker signal of a single eye was smaller than the systematic error of the version signal (averaged left and right eye signals). In the introduction we suggested that this could be due to disturbed binocular alignment. However, the prevalence of disturbed binocular alignment is only of the order of 5% (Stidwill, 1997), and could not explain the 25 to 30% that we report. There could of course be other factors affecting data quality, such as glasses, contact lenses, eyelashes, and mascara (Nyström et al., 2013). These factors may affect the data quality of the two eyes in different ways. Unfortunately, we did not take pictures of the faces of our participants and we also did not keep track of whether a participant wore make-up or had droopy eyelids.

The systematic error in the R038 experiment was not significantly larger than in the SF experiment. This is good news for the purpose of this study because there are many differences between the two experiments (evening vs afternoon; theatre vs classroom; music vs no music). Beforehand, we did not have expectations about whether these factors would affect the data quality. However, there are three other important differences between the experiments that are known to affect data quality in general:

1. Make-up may cause lower data quality. The proportion of women in the R038 experiment was slightly lower than in SF experiment (59% vs 61%). We assume that the female participants in the R038 experiment used more make-up because they were much younger (about 10 years) than the women in the SF experiment.
2. In the SF experiment we used one operator for all measurements (author NH), in the R038 experiment we used three operators, including authors GH and NH, with different levels of experience. According to Nyström et al., (2013): “the experience of operators affect the quality of data recorded with a common tower-mounted, video-based eyetracker” (p. 272).
3. The time between calibration and validation is longer in the R038 experiment (240 s) than in the SF experiment (35 s). Systematic error is known to increase as a function of time since calibration. Nyström et al., (2013) writes about this: “Offsets were greater in the second recording phase, after reading had commenced on average, around 0.2^∘^ larger than in the first recording” (p. 281).

### Using monocular eye-tracking data

Averaging left and right eye signals is an effective way to achieve a smaller variable error and in most participants (about 70%) to achieve a smaller systematic error. However, averaging cannot be applied to increase data quality in general. For example, it cannot be applied if the vergence signal (the difference between the left and right eye signal) is the topic of the study (e.g. Erkelens & Collewijn, 1985; Liversedge et al., 2006; Nuthmann & Kliegl, 2009; Zee, Fitzgibbon, & Optican, 1992).

There are also situations in which averaging LE and RE signals is technically possible but should be avoided. The reason is that on a more detailed level the left and the right eye signals may differ and averaging may affect, mask or deform these details. This is the case, for example, when eye movement dynamics are studied. Usually the study of saccade dynamics is conducted with high-end eye trackers (with a higher price, a smaller systematic error, a smaller variable error and a higher sampling rate). Without being exhaustive, we have collected some examples from the literature that show that left and right eye signals may differ. Corresponding abducting (directed away from the nose) and adducting (made in the direction of the nose) saccades may differ systematically in saccade amplitude and post saccadic drift (Kapoula, Hain, Zee, & Robinson, 1997). In addition, Collewijn et al., (1988) reported that “a marked effect of direction was found for saccades made by the eyes. Temporal saccades of one eye were significantly larger (p < 0.01) than the corresponding nasal saccades of the fellow eye for target distances up to 60 deg” (p. 165). Hooge, Nyström, Cornelissen, and Holmqvist (2015) used different types of high speed video eye trackers and report post saccadic oscillations (PSO) of corresponding abducting and adducting saccades to be different. Orquin and Holmqvist (2018) write: “even with just a slight difference in timing between the two eyes, averaging the signals could alter saccade measures such as the latency, velocity profile, and peak velocity or skew.” There are more topics in eye movement research where it is required or recommended to use data from two eyes separately (e.g., micro-saccade, 3D eye movements).

### The variable error and AOI analysis

Do researchers actually use the version signal to decrease the variable error? We assume that in fields in which small AOIs (Areas of Interest) are used, researchers use the version signal. If they choose not to, they should motivate why. A typical field with small AOIs is reading research. In Rayner, Castelhano, and Yang (2010), for example, we found: “For all participants, viewing was binocular, but only the right eye was monitored” (p. 715). In another study (Reichle, Reineberg, & Schooler, 2010) we found: “An EyeLink 1000 eye tracker monitored the gaze location of participants’ right eye during reading. The eye tracker had a spatial resolution of 30 arcmin and a 1000-Hz sampling rate” (p. 1301). These authors chose to buy an accurate eye tracker and used the eye-tracking signal of one eye. It is unclear to us why they did not choose the eye tracker signal (from left or right eye) with the smallest variable or systematic error. Probably they did not compute these values. Many other authors do not even report whether they use monocular or binocular data (e.g., Kliegl, Grabner, Rolfs, & Engbert, 2004). However, we assume (Kliegl et al., 2004) used the data of one eye, as many reading researchers do.

We suggest that in each study the authors estimate the variable and systematic errors with a calibration validation procedure for the eye-tracker signal from the separate eyes and the version signal. Hereafter, researchers should motivate why they use the eye tracker signal they use. For example, researchers may state that they use the left eye signal for ten participants, the right eye signal for seven, and the version signal for 24 participants, because the average systematic error thus obtained was 10% smaller than using the version signal for each participant (the numbers are not real, but only serve as an illustration). This may be important if the systematic error is the bottleneck for AOI analysis. Note that the same type of analysis could also lead to the choice for the version signal for all participants. We also suggest that if authors use AOIs, that they motivate the size of the AOIs and show that their data quality is good enough for this size. Orquin and Holmqvist (2018) recognize the problem of small AOIs and data quality and suggest a simulation method to determine whether the combination of AOI size, the variable and systematic errors is sufficient. A good example of this approach is found in Van der Stigchel, Hessels, van Elst, and Kemner (2017). After reporting values for the variable and systematic errors they write “While the achieved values for accuracy and precision are somewhat higher [than the manufacturer specifications], they are well within limits for fixation detection and AOI analysis” (p. 4).

## Conclusions

If a small variable error is desirable, and the difference between the left and the right eye signals is not the topic of study, one should average position data from the left and the right eye (in other words, use the version signal). If a small systematic error is desirable, one should use the signal (from left eye, right eye or version) that delivers the best accuracy. In the latter case this may cause worse precision than that of the version signal.

## Footnotes

1 Binocular eye movements can be described with a conjugate (version) and a disconjugate (vergence) component. Version is computed by averaging the left and the right eye signals; Vergence is computed by subtracting the right eye from the left eye signal.
